# Identification and Analysis of bZIP Family Genes in *Sedum plumbizincicola* and Their Potential Roles in Response to Cadmium Stress

**DOI:** 10.3389/fpls.2022.859386

**Published:** 2022-04-27

**Authors:** Zhuchou Lu, Wenmin Qiu, Kangming Jin, Miao Yu, Xiaojiao Han, Xiaoyang He, Longhua Wu, Chao Wu, Renyin Zhuo

**Affiliations:** ^1^State Key Laboratory of Tree Genetics and Breeding, Key Laboratory of Tree Breeding of Zhejiang Province, Research Institute of Subtropical Forestry, Chinese Academy of Forestry, Hangzhou, China; ^2^Faculty of Forestry, Nanjing Forestry University, Nanjing, China; ^3^Agricultural Technology Extension Centre of Dongtai, Yancheng, China; ^4^Key Laboratory of Soil Environment and Pollution Remediation, Institute of Soil Science, Chinese Academy of Sciences, Nanjing, China; ^5^Institute of Horticulture, Zhejiang Academy of Agricultural Science, Hangzhou, China

**Keywords:** bZIP gene family, *Sedum plumbizincicola*, Cd stress, expression profiles, *SpbZIP60*

## Abstract

*Sedum plumbizincicola* (Crassulaceae), a cadmium (Cd)/zinc (Zn)/lead (Pb) hyperaccumulator native to Southeast China, is potentially useful for the phytoremediation of heavy metal-contaminated soil. Basic leucine zipper (bZIP) transcription factors play vital roles in plant growth, development, and abiotic stress responses. However, there has been minimal research on the effects of Cd stress on the bZIP gene family in *S. plumbizincicola*. In this study, 92 *SpbZIP* genes were identified in the *S. plumbizincicola* genome and then classified into 12 subgroups according to their similarity to bZIP genes in *Arabidopsis.* Gene structure and conserved motif analyses showed that *SpbZIP* genes within the same subgroup shared similar intron–exon structures and motif compositions. In total, eight pairs of segmentally duplicated *SpbZIP* genes were identified, but there were no tandemly duplicated *SpbZIP* genes. Additionally, the duplicated *SpbZIP* genes were mainly under purifying selection pressure. Hormone-responsive, abiotic and biotic stress-responsive, and plant development-related *cis*-acting elements were detected in the *SpbZIP* promoter sequences. Expression profiles derived from RNA-seq and quantitative real-time PCR analyses indicated that the expression levels of most *SpbZIP* genes were upregulated under Cd stress conditions. Furthermore, a gene co-expression network analysis revealed that most edge genes regulated by hub genes were related to metal transport, responses to stimuli, and transcriptional regulation. Because its expression was significantly upregulated by Cd stress, the hub gene *SpbZIP60* was selected for a functional characterization to elucidate its role in the root response to Cd stress. In a transient gene expression analysis involving *Nicotiana benthamiana* leaves, *SpbZIP60* was localized in the nucleus. The overexpression of *SpbZIP60* enhanced the Cd tolerance of transgenic *Arabidopsis* plants by inhibiting ROS accumulation, protecting the photosynthetic apparatus, and decreasing the Cd content. These findings may provide insights into the potential roles of the bZIP family genes during the *S. plumbizincicola* response to Cd stress.

## Introduction

Heavy metal pollution has become a global environmental problem (Ali et al., 2013). Cadmium (Cd) is a major heavy metal pollutant that is released into the environment because of human industrial and agricultural production activities (Tchounwou et al., 2012). Cd contamination leads to decreased soil quality and suppressed crop production. Heavy metal stress results in changes to various physiological and metabolic processes. For example, the expression of many genes is induced in plants under heavy metal stress conditions, and the upregulated expression of stress-responsive genes, which is usually mediated by transcription factors, may increase plant survival rates (Yao et al., 2018; Zhang et al., 2020; Xu et al., 2021b).

The basic leucine zipper (bZIP) family is one of the largest and most diverse transcription factor families in eukaryotes (Pérez-Rodríguez et al., 2010). These transcription factors contain a highly conserved bZIP domain with two different functional regions, one of which is a sequence-specific DNA-binding alkaline region (N-x7-R/K-x9), whereas the other is a leucine zipper consisting of several heptapeptide repeats comprising Leu or other large hydrophobic amino acids (e.g., Ile, Val, Phe, or Met) that influence dimerization specificity (Jakoby et al., 2002; Nijhawan et al., 2008). The bZIP gene family was first identified and classified in *Arabidopsis* at the genome-wide level (Jakoby et al., 2002). The current study is related to earlier research, during which 78 *AtbZIP* genes were identified and divided into 13 subclasses (A–M) (Dröge-Laser et al., 2018). Additionally, analyses of the bZIP gene family in diverse species resulted in the identification of 64 genes in cucumber (Corrêa et al., 2008), 85 genes in rice (Nijhawan et al., 2008), 86 genes in poplar (Zhao et al., 2021), 96 genes in buckwheat (Liu et al., 2019b), 112 genes in apple (Zhao et al., 2016), 125 genes in maize (Wei et al., 2012), and 160 genes in soybean (Zhang et al., 2018).

There is considerable evidence that bZIP transcription factors in plants play crucial roles in various biological processes, including seed maturation (Izawa et al., 1994), organ differentiation (Pautler et al., 2015), photomorphogenesis (Huang et al., 2012), and floral development (Abe et al., 2005; Muszynski et al., 2006). They also contribute to responses to various abiotic stresses, including salinity (Bi et al., 2021), drought (Wang et al., 2017; Tu et al., 2020), heat (Deng et al., 2011; Liu et al., 2012), osmotic stress (Xu et al., 2013), and oxidative stress (Choi et al., 2021). Most of these responses are abscisic acid (ABA) signal transduction-dependent processes (Banerjee and Roychoudhury, 2017). As a key member of the ABA signal transduction pathway, bZIP proteins are activated by kinases, such as SnRK2, and then bind to an ABA-responsive element (ABRE) to regulate the expression of downstream genes. In rice, *OsbZIP46* positively regulates ABA signal transduction and drought stress tolerance (Tang et al., 2012). The stress-induced expression of the activated form of *AtbZIP17* protects *Arabidopsis* from salt stress (Liu et al., 2008). In poplar, a loss-of-function mutation to *PtabZIP1* enhances lateral root formation under osmotic stress conditions (Dash et al., 2017). As the most dangerous pollutant, heavy metals have been regarded as new stress factors.

Similar to other abiotic stress responses, there has been increasing interest in the relationship between bZIP transcription factors and heavy metal stress responses. The *BjCdR15/TGA3* transcription factor gene encodes an important regulator of Cd uptake by roots and the subsequent long-distance root-to-shoot transport. The overexpression of this gene in *Arabidopsis* and tobacco enhances Cd tolerance and accumulation. This is related to the regulation of the synthesis of phytochelatin synthase and the expression of several metal transporter genes (Farinati et al., 2010). In *Arabidopsis*, ABI5 (ABA-Insensitive 5), which is a central ABA signaling molecule, represses Cd accumulation in plants by physically interacting with MYB49 and preventing it from binding to the downstream genes *bHLH38*, *bHLH101*, *HIPP22*, and *HIPP44*, resulting in the inactivation of *IRT1* and decreased Cd uptake (Zhang et al., 2019). The subgroup F bZIP transcription factors *AtbZIP19* and *AtbZIP23* are Zn sensors that regulate *Arabidopsis* responses to Zn deficiency *via* the binding between Zn^2+^ ions and their Zn sensor motif (Assunção et al., 2010; Lilay et al., 2019, 2021). Thus, the bZIP transcription factors appear to participate in plant responses to heavy metal stress.

Current research on the heavy metal homeostasis in plants primarily focuses on model plants or crop plants. Hyperaccumulator plants are valuable research materials because of their potential utility for remediating heavy metal-contaminated soil. Moreover, they are useful for investigating plant adaptation and evolution in extreme environments. The Cd, Pb, and Zn hyperaccumulator *Sedum plumbizincicola* (Wu et al., 2013), which is also known as the hyperaccumulating ecotype of *S. alfredii* (Yang et al., 2002), can tolerate, transport, and accumulate large amounts of Cd (Li et al., 2018), with a shoot Cd concentration as high as 9,000 mg/kg (Yang et al., 2004). Its efficient Cd absorption, transport, and detoxification systems are necessary for its growth in highly contaminated soils. Some genes related to Cd absorption, resistance, and hyperaccumulation, such as *SpHMA3* (Liu et al., 2017), *SpMTL* (Peng et al., 2017), *SaNramp6* (Chen et al., 2017; Lu et al., 2020), *SaCAX2* (Zhang et al., 2016), *SaHsfA4c* (Chen et al., 2020), *SaCAD* (Qiu et al., 2018), *SaREF* (Liu et al., 2016), and *SaPCR2* (Lin et al., 2020), have been characterized. However, there has yet to be a systematic analysis of the transcription factor families (e.g., bZIP) in *S. plumbizincicola* to clarify their roles in response to heavy metal stress.

In this study, we identified 92 bZIP genes in the *S. plumbizincicola* genome and then analyzed their structures, motifs, *cis*-acting elements, and phylogenetic relationships. On the basis of RNA sequencing (RNA-seq) and quantitative real-time PCR (qRT-PCR) methods, we explored their expression profiles in response to Cd stress. Furthermore, the bZIP60 function related to plant responses to Cd stress was investigated. The results of this study will be useful for the future functional characterization of the *SpbZIP* genes in terms of their roles during plant responses to Cd stress.

## Materials and Methods

### Identification of the Basic Leucine Zipper Family Genes in *Sedum plumbizincicola*

To identify all members of the bZIP gene family in *S. plumbizincicola*, HMMER3.0 was used to screen for candidate proteins in the *S. plumbizincicola* genome database (unpublished work) on the basis of the Hidden Markov Model profile of the bZIP domain (PF00170).^1^ A BLASTP search was performed using 78 *Arabidopsis* protein sequences that were annotated according to previously published methods from TAIR.^2^ Subsequently, Pfam, SMART,^3^ and CDD^4^ were used to confirm the presence of the bZIP domain in candidate proteins. All putative bZIP genes were named according to their homologs in *Arabidopsis*. The encoded protein sequences were analyzed using the online tool ProtParam^5^ to predict the amino acid composition, molecular weight, and isoelectric point (Gasteiger et al., 2005). Additionally, PSORT prediction^6^ was used to predict the subcellular localization of the proteins.

### Multiple Sequence Alignment and Phylogenetic Analysis

ClustalX2 was used to align the full-length SpbZIP and AtbZIP amino acid sequences. Phylogenetic trees were constructed using the maximum-likelihood criteria in MEGA 5.0, with 1,000 bootstrap replicates. The identified *SpbZIP* genes were divided into different groups according to the *AtbZIP* classification scheme. The phylogenetic tree was visualized using iTOL.^7^

### Analysis of *cis*-Acting Elements in *SpbZIP* Promoters

The *cis*-acting elements in the promoter region 2 kb upstream of the *SpbZIP* genes were identified and then submitted to the PlantCARE database^8^ (Lescot et al., 2002). The position of the identified elements was graphically displayed using the TBtools software.^9^

### Analysis of *SpbZIP* Gene Structures and Encoded Motifs

The exon/intron structure of *SpbZIP* genes was analyzed and displayed using the GSDS platform.^10^ The conserved motifs in the SpbZIP proteins were identified using the MEME program (version 5.0.5),^11^ with the following parameters: optimum motif width range of 6–50 amino acid residues and a maximum of 22 motifs (Bailey and Elkan, 1994).

### Synteny Analysis and Chromosomal Distribution of *SpbZIP* Genes

The default parameters of the Multiple Collinearity Scan (MCScanX) toolkit were used to analyze gene duplication events (Wang et al., 2012). Diagrams were generated using the Circos program (version 0.69)^12^ (Krzywinski et al., 2009). Non-synonymous (ka) and synonymous (ks) substitutions in each duplicated *SpbZIP* gene were calculated using KaKs_Calculator 2.0 (Wang et al., 2010).

### Plant Materials and Cd Stress Treatments

*Sedum plumbizincicola* plants were collected from an old Pb/Zn mine in Huiping town, Quzhou city, Zhejiang province, China. The shoots from a single genotype were asexually propagated and cultivated in water in an artificial climate chamber at 25°C with a 16-h light/8-h dark cycle. The plants were grown in a half-strength Hoagland solution for about 4 weeks. Similarly growing plants were then treated with 400 μM CdCl_2_. The roots, stems, and leaves were sampled at 0, 0.5, 2, 4, 8, and 12 h after the Cd stress treatment. Three biological replicates were collected for all samples.

### *SpbZIP* Expression Profiles in Response to Cd Stress

The Total RNA Purification kit (NORGEN, Thorold, ON, Canada) was used to extract total RNA from the roots, stems, and leaves. First-strand cDNA was generated using PrimeScript™ RT Master Mix (TaKaRa, Dalian, China). The qRT-PCR analysis was performed in triplicate using the 7,300 Real-Time PCR System (Applied Biosystems, CA, United States) and the SYBR^®^ Premix Ex Taq™ reagent (TaKaRa, Dalian, China). Gene-specific primers were designed using the “Genes” module of the SPDE software (Xu et al., 2021a). The UBC gene was selected as the internal reference (Sang et al., 2013). Primers used are listed in Supplementary Table 5. Relative expression levels were calculated according to the 2^–ΔΔCT^ method (Livak and Schmittgen, 2001). The FPKM values for the *SpbZIP* genes were derived from the RNA-seq data (Han et al., 2016). Expression values were normalized *via Z*-score normalization. An expression profile heatmap was generated using the pheatmap package in R (4.0.2).

### *SpbZIP* Co-expression Regulatory Network

The weighted gene co-expression network analysis (WGCNA) R package was used to construct a co-expression regulatory network on the basis of the expression profiles of differentially expressed genes under Cd stress conditions (Han et al., 2016). The *SpbZIP* genes among the co-expressed genes with strong interconnections were designated as hub genes. The Pearson’s correlation coefficient threshold was set as 0.40 according to the FPKM values for each gene pair using the R (version 4.0.2) program (Han et al., 2016). We screened for co-expression edge genes associated with the *SpbZIP* hub genes and performed Gene Ontology (GO) analyses using the Gene Annotation Software for Plants (GFAP) (Xu et al., 2022). Subsequently, we classified the related genes according to their functions and visualized the relationships between nodes and edges using Cytoscape (version 3.6.1).

### Subcellular Localization of *SpbZIP60*

The *SpbZIP60* coding sequence without the stop codon was fused to the mGFP-encoding sequence in the pCAMBIA1302 expression vector using the ClonExpress II One Step Cloning Kit (Vazyme, Nanjing, China). *Agrobacterium tumefaciens* GV3101 cells were transformed with the recombinant plasmid, which was then transferred into healthy *Nicotiana benthamiana* leaves for a transient gene expression analysis; the empty vector was used as a control. After co-culturing for 3 days, the leaves were soaked in a 4,6-diamidino-2-phenylindole (DAPI) staining solution to visualize nuclear DNA. The LSM 710 confocal laser-scanning microscope (Zeiss, Germany) was used to detect the fluorescence of the fusion protein.

### Ectopic Expression of *SpbZIP60* in *Arabidopsis* and Cd Treatment

The *SpbZIP60* coding sequence was amplified by PCR and inserted into the pCAMBIA1300 vector. The recombinant plasmid was inserted into *Arabidopsis* (Col-0) plants *via A. tumefaciens* (EHA105)-mediated transformation (Zhang et al., 2006). The T_3_ homozygous transgenic lines and wild-type (WT) plants were grown in a half-strength Hoagland solution. The seedlings were transferred to a solution containing 30 μM CdCl_2_ after 4 weeks and grown for 7 days. The roots of the treated seedlings were immersed in a 10-mM EDTA solution for 0.5 h to remove Cd from the surface. The samples were dried and then digested with a solution comprising HNO_3_ and perchloric acid (9:1 v/v) at 120–200°C in a microwave-accelerated reaction system (CEM, Matthews, NC, United States). The Cd content was determined using the 7500a inductively coupled plasma mass spectrometry system (Agilent, Santa Clara, CA, United States). Previously described 3,3’-diaminobenzidine (DAB) and nitroblue tetrazolium (NBT) staining methods were used to reveal the presence of H_2_O_2_ and O_2_^–^ *in situ* (Chen et al., 2020). The chlorophyll content was measured according to an acetone ethanol extraction method (Li et al., 2000). Chlorophyll fluorescence was analyzed using the Dual-PAM-100 system (Walz, Effeltrich, Germany); the parameters were set, and the data were analyzed as previously described (Su et al., 2020).

## Results

### Identification and Characterization of Putative Basic Leucine Zipper Transcription Factors

Following a search of the *S. plumbizincicola* genome database using HMMER3.0, the identified candidate sequences were examined using CDD, Pfam, and SMART to confirm the presence of the bZIP domain (*E*-value < 1e^–5^). A total of 92 non-redundant genes were identified as bZIP genes in the *S. plumbizincicola* genome. They were named according to the corresponding *Arabidopsis* homologs. The subsequent analysis indicated that the SpbZIP proteins comprise 117–707 amino acids (average of 303 amino acids), with a molecular weight of 13.7–77.3 kDa (average of 33.7 kDa) and a predicted isoelectric point of 5.05–10.26 (average of 7.02). Most of the identified SpbZIP proteins were predicted to localize in the nucleus, which is a characteristic of transcription factors (Supplementary Table 1).

### Phylogenetic Analysis of *SpbZIP* Genes

**FIGURE 1:**
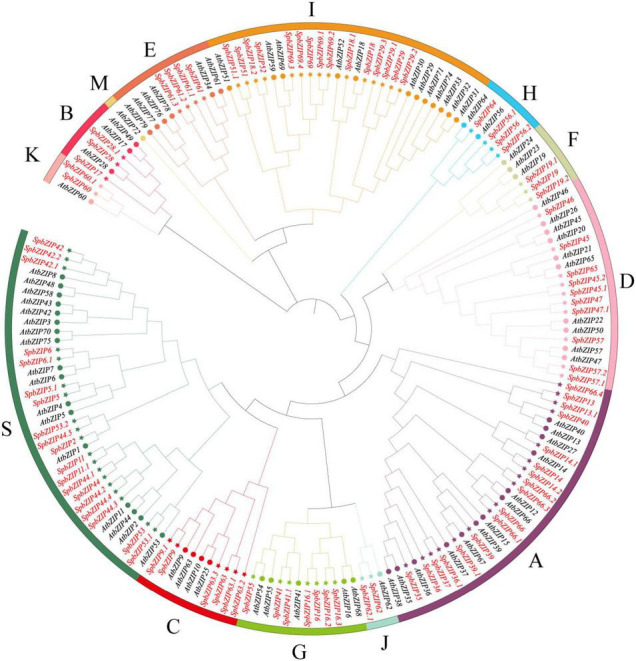
Phylogenetic relationships among the bZIP genes from *Sedum plumbizincicola* and *Arabidopsis*. The phylogenetic tree was constructed on the basis of the alignment of *S. plumbizincicola* and *Arabidopsis* bZIP proteins according to the maximum-likelihood method, with 1,000 bootstrap replicates.

To classify the *SpbZIP* genes into subgroups and elucidate the evolutionary relationships between *S. plumbizincicola* and *Arabidopsis* genes, we constructed an unrooted phylogenetic tree using the maximum-likelihood method and the protein sequences encoded by 78 *AtbZIP* genes and the 92 identified *SpbZIP* genes (Figure 1). On the basis of the phylogenetic tree, the *SpbZIP* genes were divided into 12 of 13 subgroups; the exception was subgroup M. There were no individual clades among the *SpbZIP* genes, suggesting that they were relatively conserved. Similar to the *Arabidopsis* homologs, most of the *SpbZIP* genes were classified into subgroups S and A. Subgroups J and K had the fewest genes, each with only two *SpbZIP* genes.

### *SpbZIP* Gene Structure and Protein Motif Composition

**FIGURE 2:**
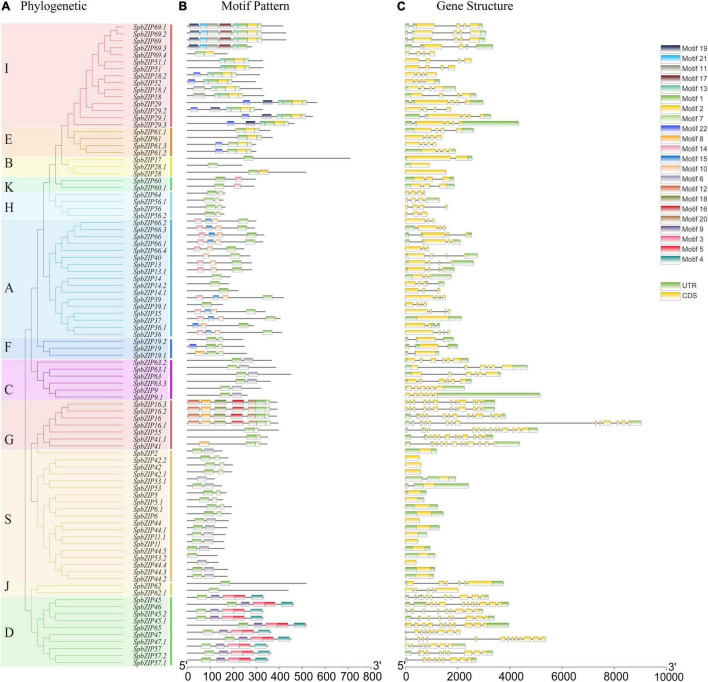
Phylogenetic relationships, motif compositions, and gene structures of *SpbZIP* genes in *S. plumbizincicola*. **(A)** Phylogenetic analysis of *S. plumbizincicola* bZIP family members. **(B)** All conserved motifs in the SpbZIP proteins were identified using the MEME program. Different motifs are highlighted with different colored boxes (numbered 1–22). **(C)** Gene structures. Exons and 5’/3’ untranslated regions are indicated by green and yellow bars, respectively, whereas gray lines represent introns.

To gain insights into the structures of *SpbZIP* genes, their introns and exons were analyzed. Of the *SpbZIP* genes in subgroup S, 20 (21.7%) lacked introns. In contrast, three (3.3%) and seven (7.6%) genes contained one and two introns, respectively. Three or more introns were detected in 62 genes (68.5%) (Figure 2C). An examination using the MEME online program detected 22 conserved motifs in the SpbZIP proteins. The conserved motifs comprised 20–50 amino acids. Details regarding the 22 putative motifs are provided in Supplementary Table 2. Motif 1 (leucine zipper region of bZIP) was identified as the core conserved domain. A few subgroup-specific motifs were identified, including motifs 10 and 15 (subgroup A) and motifs 11, 12, and 16 (subgroup G). Most of the SpbZIP proteins in the same subgroup in the phylogenetic tree had common motifs, indicating a close evolutionary relationship and a high degree of conservation.

### Chromosomal Locations and Collinearity Analysis of *SpbZIP* Genes

**FIGURE 3:**
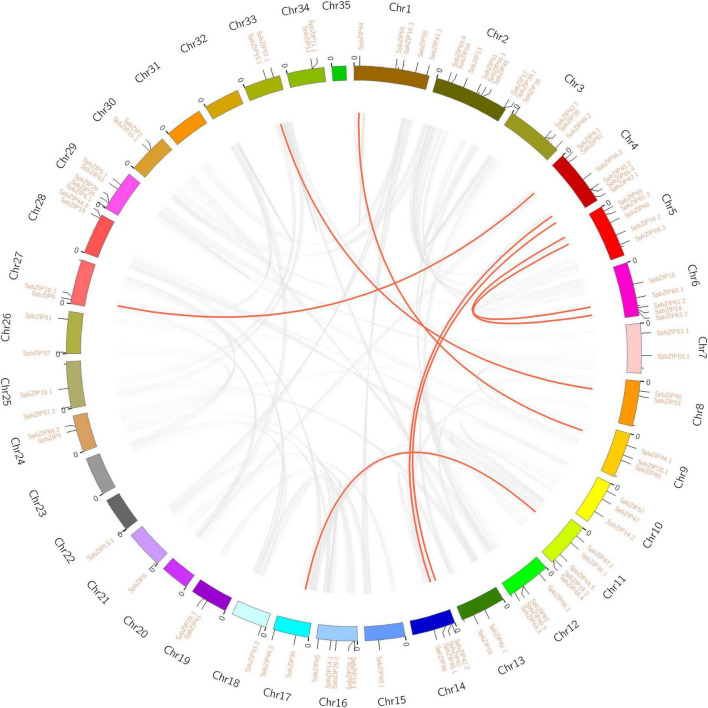
Genome location and synteny of bZIP genes in *S. plumbizincicola*. Gray lines indicate syntenic blocks in the *S. plumbizincicola* genome, whereas the red lines between chromosomes indicate segmentally duplicated gene pairs.

The 92 *SpbZIP* genes were distributed unequally among 30 *S. plumbizincicola* chromosomes (Figure 3). Segmental duplications of multiple genes are caused by chromosomal rearrangements (Yu et al., 2005), whereas tandem duplications, which mainly occur in the recombination region of chromosomes, usually result in the formation of a cluster of genes with similar sequences and functions (Ramamoorthy et al., 2008). During evolution, segmental and tandem duplications are the two main drivers of the expansion of plant gene families. In the *S. plumbizincicola* genome, eight segmental duplication events involving 16 *SpbZIP* genes (i.e., 17.4% of the *SpbZIP* genes) were detected. Among the segmentally duplicated gene pairs, *SpbZIP42.1*/*SpbZIP42* and *SpbZIP45.2*/*SpbZIP45.1* were distributed on chromosomes 4 and 14, respectively, whereas *SpbZIP60*/*SpbZIP60.1* and *SpbZIP61.3*/*SpbZIP61.2* were distributed on chromosomes 5 and 6, respectively. Additionally, *SpbZIP36.1*/*SpbZIP36*, *SpbZIP53*/*SpbZIP53.1*, *SpbZIP44*/*SpbZIP44.1*, and *SpbZIP52/SpbZIP18.2* resulted from gene duplication events. Of these gene pairs, six were assigned to subgroup S. Furthermore, none of the genes were the result of tandem duplications. Thus, we speculated that segmental duplications were important for the expansion of the *SpbZIP* family in *S. plumbizincicola*. Moreover, the Ka/Ks ratios for all eight duplicated *SpbZIP* gene pairs were less than 0.5 (Supplementary Table 3), indicating that the *SpbZIP* family paralogs were primarily under purifying selection.

**FIGURE 4:**
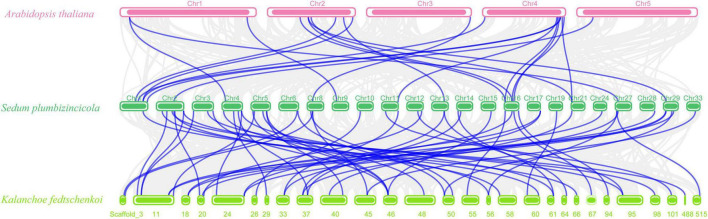
Synteny between *SpbZIP* genes and genes in other species (*Arabidopsis* and *K. fedtschenkoi*). Gray lines in the background represent collinear blocks in *S. plumbizincicola* and the other species, whereas blue lines indicate syntenic bZIP gene pairs.

Next, we created two comparative syntenic maps of the association between *S. plumbizincicola* and *Arabidopsis* or *Kalanchoe fedtschenkoi*, which is a representative Crassulaceae plant species, to further clarify the origin and evolution of the *S. plumbizincicola* bZIP family (Figure 4). A total of 15 *SpbZIP* genes had a syntenic relationship with 17 and 48 genes in *Arabidopsis* and *K. fedtschenkoi*, respectively (Supplementary Table 4). Additionally, 20 orthologous gene pairs were detected between *S. plumbizincicola* and *Arabidopsis*, which was fewer than the 54 orthologous gene pairs between *S. plumbizincicola* and *K. fedtschenkoi*. There were more collinear gene pairs between *S. plumbizincicola* and *K. fedtschenkoi* than between *S. plumbizincicola* and *Arabidopsis*, which is in accordance with the fact *S. plumbizincicola* is phylogenetically closer to *K. fedtschenkoi* than to *Arabidopsis*. Some collinear gene pairs (involving 11 *SpbZIP* genes) among all three species were identified, implying that the orthologous gene pairs may have existed before ancestral divergence. These orthologous genes were also under intense purifying selection.

### Analysis of *cis*-Acting Elements in *SpbZIP* Promoters

**FIGURE 5:**
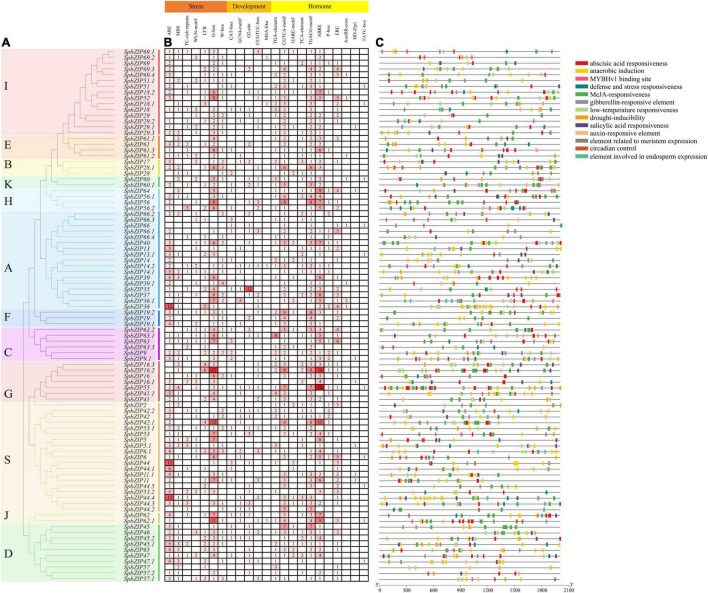
Analysis of *cis*-acting elements in the *SpbZIP* promoter region. **(A)** Phylogenetic analysis of *SpbZIP* genes. **(B)** The number of each *cis*-acting element in the promoter region (2 kb upstream of the translation start site) of *SpbZIP* genes. **(C)** Distribution of related *cis*-acting elements in *SpbZIP* promoters.

To clarify the regulatory mechanisms underlying *SpbZIP* expression, the *cis*-acting elements in the promoter sequences were analyzed using PlantCARE. The identified *cis*-acting elements (Figure 5) were divided into three categories (stress-responsive, plant development-related, and phytohormone responsive). The following seven abiotic stress-responsive elements were detected: ARE (important for anaerobic induction), MBS (MYB-binding site associated with drought-inducible expression), TC-rich repeat (stress-responsive element), WUN-motif (wound-responsive element), LTR (low temperature-responsive element), G-box, and W-box. At least one stress-responsive *cis*-acting element was detected in the promoter of all *SpbZIP* genes, with the exception of *SpbZIP66*, reflecting the importance of *SpbZIP* expression for plant responses to various abiotic stresses. Among the phytohormone-responsive *cis*-acting elements, ABRE was the most common, with 251 ABREs detected in 72 *SpbZIP* promoters (enrichment level of 3.49), followed by MeJA-responsive *cis*-acting elements (TGACG-motif and CGTCA-motif) (enrichment level of 2.49).

### *SpbZIP* Expression Profiles Under Cd Stress Conditions

**FIGURE 6:**
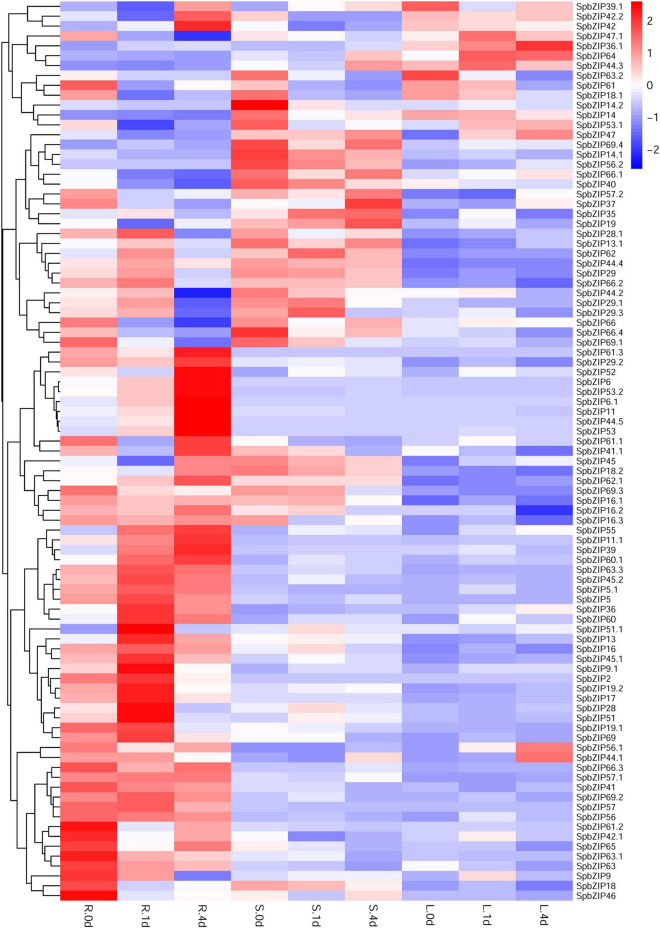
Expression profiles of *SpbZIP* genes in plant tissues under Cd stress conditions. Gene expression data at 0, 1, and 4 days after the 400 μM CdCl_2_ treatment were retrieved from an RNA-seq database and visualized using R (version 4.0.2). Expression levels are indicated by a gradient from low (blue) to high (red). L, S, and R represent leaves, stems, and roots, respectively.

**FIGURE 7:**
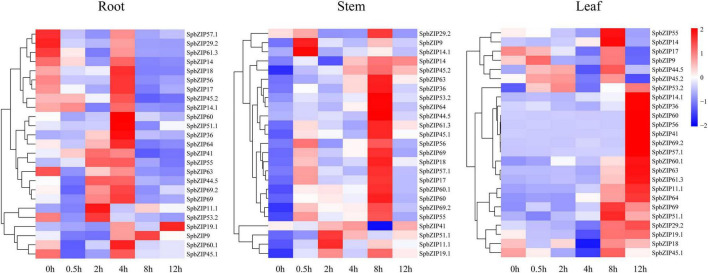
Expression profiles of hub *SpbZIP* genes in different tissues of *S. plumbizincicola* soon after the exposure to Cd stress.

We used our previously published RNA-seq data to determine *SpbZIP* expression patterns (Han et al., 2016), which were revealed in terms of FPKM values, in the roots, stems, and leaves. The *SpbZIP* expression trends in the roots during the Cd treatment period were divided into four categories (Figure 6). The expression levels of 32 *SpbZIP* genes gradually decreased or increased over the entire treatment period. In contrast, the expression levels of 18 genes peaked at 1 day after initiating the Cd treatment. However, the genes whose expression in the roots was not induced by Cd stress had upregulated or downregulated expression levels in the stems (27) or leaves (8) in response to the Cd treatment. These results suggested that SpbZIP transcription factors may play a major role in the roots as part of the initial response to Cd stress. Transcription factors often rapidly respond to environmental cues. We further shortened and refined the treatment time and then performed qRT-PCR analysis to investigate the expression levels of 25 hub genes selected from the co-expression network. As expected, for most of the *SpbZIP* genes, the expression levels peaked earlier in the roots (4 h) than in the stems (8 h) and leaves (12 h) (Figure 7).

### *SpbZIP* Co-expression Network

**FIGURE 8:**
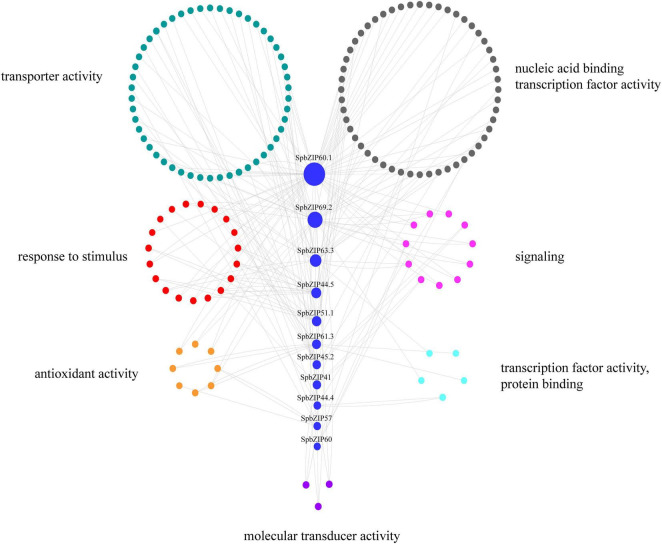
*SpbZIP* gene co-expression network. The genes are divided on the basis of the following seven GO terms, which are represented by different colors: transporter activity, nucleic acid binding transcription factor activity, response to stimulus, signaling, antioxidant activity, protein binding transcription factor activity, and molecular transducer activity.

To further clarify the regulatory effects of bZIP family members on the expression of Cd-responsive genes, a co-expression regulatory network was constructed on the basis of the expression profiles of differentially expressed genes under Cd stress conditions determined in an earlier transcriptome analysis, in which 11 *SpbZIP* genes were annotated as hub genes. The nodes associated with hub genes were clustered according to functional categories, which reflected their association with metabolic processes, cellular activities, membranes, cells, binding, and catalytic activities (Supplementary Table 6). The Cd-responsive gene co-expression network had 189 nodes (Figure 8). The major categories included transcription factor (59 edges), transporter activity (52 edges), stimulus-response (43 edges), signaling (19 edges), and antioxidant activity (8 edges). The hub gene *SpbZIP60.1* was associated with the most nodes (59), including 19 transcription factor nodes, 12 transporter activity nodes, 4 stimulus-response nodes, and 4 signaling nodes, followed by *SpbZIP69.2* (34 nodes) and *SpbZIP63.3* (21 nodes). Accordingly, in response to Cd stress, SpbZIP transcription factors appear to regulate the expression of downstream genes associated with diverse functions.

### *SpbZIP60* Was Localized in the Nucleus

**FIGURE 9:**
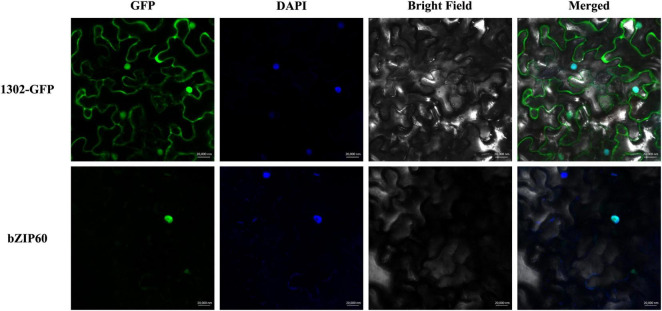
Subcellular localization of SpbZIP60. The SpbZIP60-GFP fusion construct and the GFP gene driven by the CaMV 35S promoter were transiently expressed in tobacco. The nucleus was visualized using the DAPI staining solution.

In this study, *SpbZIP60* was one of the hub genes in the co-expression regulatory network, and its expression level was significantly upregulated in the roots during the Cd stress treatment. Hence, the subcellular localization of SpbZIP60 was analyzed to elucidate the potential functions of bZIP transcription factors in *S. plumbizincicola*. The control GFP signal was distributed throughout the cell, whereas the fluorescence of the SpbZIP60-mGFP fusion protein was detected only in the nucleus (Figure 9). Thus, SpbZIP60 likely functions as a nuclear protein that regulates transcription.

### Overexpression of *SpbZIP60* Enhanced the Cd Tolerance of *Arabidopsis*

To further explore the function of SpbZIP60 under Cd stress conditions, transgenic *Arabidopsis* plants overexpressing *SpbZIP60* were generated. The T_0_ transgenic lines were verified by PCR using genomic DNA as the template. After analyzing the *SpbZIP60* expression levels by semi-RT-PCR, the transgenic lines were cultivated to produce the homozygous T_3_ lines used for the subsequent analyses (Supplementary Figure 1).

**FIGURE 10:**
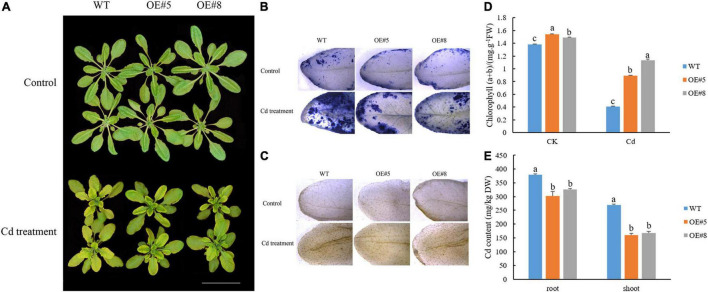
Effects of Cd stress treatments on the growth of *SpbZIP60*-overexpressing *Arabidopsis* plants. **(A)** Phenotypes of *SpbZIP60*-overexpressing transgenic lines and wild-type (WT) plants under normal conditions or in response to the Cd treatment. Bar = 5 cm. **(B)** NBT staining results. **(C)** DAB staining results. **(D)** Chlorophyll contents of the WT and transgenic lines before and after the Cd treatment. **(E)** Cd contents of the WT and transgenic lines. Bars represent the mean ± standard deviation (SD) of at least three independent biological replicates. Significant differences according to a one-way analysis of variance are denoted as follows: *p* < 0.05 (Duncan’s test).

Leaf chlorosis and damages to the photosynthetic apparatus are observable symptoms of Cd toxicity. The degree of chlorosis in leaves at 7 days after initiating the Cd stress treatment was higher in the WT plants than in the *SpbZIP60*-overexpressing plants (Figure 10A). Histochemical staining revealed that less H_2_O_2_ and O_2_^–^ accumulated in the transgenic *Arabidopsis* lines (OE#5 and OE#8) than in the WT control following the Cd treatment (Figures 10B,C). Meanwhile, the total chlorophyll content of the *SpbZIP60*-overexpressing plants where significantly higher than those of WT (Figure 10D). Chlorophyll fluorescence properties, which reflect the photochemical processes of PSII, are a useful indicator of the effects of heavy metal stress, especially Cd stress, on the photosynthetic apparatus. In the WT *Arabidopsis* plants, the Fv/Fm decreased, which was indicative of photoinhibition. Moreover, the inactivation or destruction of PSII resulted in an increase in the initial fluorescence (F_0_). Additionally, the relative PSII electron transport rate was higher in the *SpbZIP*-overexpressing plants than in the WT plants (Supplementary Table 7). These results suggested that in response to Cd stress, the photosynthetic apparatus was damaged less in the *SpbZIP60*-overexpressing plants than in the WT plants. Next, we analyzed the Cd concentrations in hydroponically grown *SpbZIP60*-overexpressing lines. The Cd concentrations in the leaves and roots decreased substantially in the transgenic lines (Figure 10E). Therefore, SpbZIP60 significantly decreased the Cd concentration in the roots of the transgenic *Arabidopsis* plants, likely by inhibiting Cd uptake.

## Discussion

*Sedum plumbizincicola* has undergone long-term evolution and natural selection in heavy metal-contaminated soil (Wu et al., 2013; Yang et al., 2017). The *S. plumbizincicola* proteins involved in the absorption, transport, sequestration, and detoxification of heavy metals have been thoroughly studied, especially the heavy metal transporters (Liu et al., 2016, 2017, 2019a; Peng et al., 2017; Chen et al., 2020; Zhu et al., 2022). However, systematic analyses of the transcriptional regulation of the genes encoding these proteins have not been conducted. Transcription factors in the bZIP family modulate various physiological processes and abiotic stress responses (Corrêa et al., 2008). Thus, characterizing the *S. plumbizincicola* bZIP family is critical for clarifying the mechanism underlying the responses of *S. plumbizincicola* plants to environmental factors, especially heavy metal stress.

In this study, we conducted a genome-wide analysis of the *S. plumbizincicola* bZIP transcription factor family and explored the potential functions in response to Cd stress. On the basis of the encoded motifs, 92 *SpbZIP* genes were identified in the *S. plumbizincicola* genome. The number of bZIP genes in *S. plumbizincicola* is higher than that in some plant species but lower than that in other plant species (Corrêa et al., 2008; Nijhawan et al., 2008; Wei et al., 2012; Zhao et al., 2016, 2021; Dröge-Laser et al., 2018; Zhang et al., 2018; Liu et al., 2019b). We then divided the 92 *SpbZIP* genes into 12 subgroups after comparing the encoded protein sequences with the corresponding sequences in *Arabidopsis*. The classification of the bZIP genes was relatively consistent between *S. plumbizincicola* and *Arabidopsis*. However, *AtbZIP72* was included in a separate clade (subgroup M), which lacked *SpbZIP* genes, suggesting that this clade is specific to *Arabidopsis*. In the phylogenetic tree constructed in this study, there were no branches that were exclusive to *S. plumbizincicola*, suggesting the *SpbZIP* genes are evolutionarily conserved (Figure 1). Moreover, genes belonging to the same subgroup were revealed to share similar gene structures and encode common motifs (Figure 2). For example, subgroup S consisted of small proteins encoded by genes lacking introns, which is in accordance with the results of earlier studies (Dröge-Laser et al., 2018; Wang et al., 2021).

Tandem and segmental duplication events are crucial for the expansion of gene families and the diversification of gene functions, which have enabled plants to adapt to environmental conditions (Cannon et al., 2004). We detected eight pairs of segmentally duplicated genes on 11 chromosomes, but no tandemly duplicated genes. Therefore, the expansion of the bZIP gene family in *S. plumbizincicola* was mainly the result of segmental duplications. The calculated Ka/Ks ratios for all gene pairs were less than 0.5, implying these genes might have experienced strong purifying selection pressure during evolution. Furthermore, we analyzed the collinearity between the *SpbZIP* genes and genes in *Arabidopsis* and *K. fedtschenkoi*. There were more collinear gene pairs between *S. plumbizincicola* and *K. fedtschenkoi*, which has a relatively close evolutionary relationship with *S. plumbizincicola*, than between *S. plumbizincicola* and *Arabidopsis*. A comparison between *S. plumbizincicola* and *Arabidopsis* detected 20 orthologous pairs of bZIP genes. As putative orthologs of *SpbZIP19.1*, both AT4G35040.1 (*AtbZIP19*) and AT2G16770.1 (*AtbZIP23*), which belong to subgroup F, encode Zn sensors that contain a motif that binds Zn^2+^ ions, enabling them to regulate plant responses to zinc deficiency (Lilay et al., 2021). Additionally, the following four G-box-binding factors (GBFs) were identified: GBF1 (SpbZIP41.1/AT4G36730.1), GBF2 (SpbZIP55/AT4G01120.1), GBF3 (SpbZIP55/AT2G46270.1), and GBF6 (SpbZIP16.3 and SpbZIP44.4/AT4G34590.1). Previous research indicated that GBFs participate in abiotic stress responses (Sun et al., 2015). For example, the expression of *AtGBF3* induces drought and pathogen stress tolerance by activating ABA-mediated signaling (Ramegowda et al., 2017; Dixit et al., 2019). Interestingly, the promoter of *SpbZIP55*, which is orthologous to *AtGBF3*, was revealed to contain the most ABREs among the examined *SpbZIP* genes, suggesting that *SpbZIP55* may also be related to ABA signaling and stress responses.

We further explored the *SpbZIP* expression patterns in response to Cd stress. Most of the *SpbZIP* genes were responsive to Cd stress, especially in the roots. This finding may be related to the fact that plants first perceive Cd stress in the roots, which take up Cd from the soil. The Cd is then transported to the stems and leaves. Therefore, the response to Cd stress will likely be greater in the roots than in the other plant tissues (Pan et al., 2019). Transcription factors may regulate metal ion transport in the stem. For example, in *Brassica juncea*, BjCdR15/TGA3 is a transcription factor that is crucial for the regulation of Cd uptake by the roots and the root-to-shoot transport of Cd (Farinati et al., 2010). Moreover, bZIP genes encode transcription factors that respond rapidly to stimuli. A co-expression regulatory network analysis is useful for identifying closely co-regulated and functionally related genes or genes affecting the same signaling pathway or physiological process. To identify the core *SpbZIP* genes responsive to Cd stress, we constructed a co-expression network and identified 11 hub *SpbZIP* genes that are co-expressed, with strong interconnections to edges (Han et al., 2016). These genes may encode proteins that sense specific signals, respond to stimuli, regulate the expression of other transcription factor genes, and ultimately affect metal transport or oxidative elimination.

The hub gene *SpbZIP60* was selected for functional analysis because its expression was observed to be upregulated by Cd stress. The overexpression of *SpbZIP60* in transgenic *Arabidopsis* resulted in increased Cd tolerance. More specifically, the photosynthetic apparatus was damaged more in the WT plants than in the transgenic plants following the Cd treatment. Furthermore, Cd accumulated less in the transgenic plants than in the WT controls. These results indicate that SpbZIP60 may affect the uptake or transport of Cd. However, it is unclear whether the increased Cd resistance is also the result of enhancements to other detoxification-related processes. The *Chlamydomonas* bZIP transcription factor BLZ8 confers oxidative stress tolerance by inducing a carbon-concentrating mechanism (Choi et al., 2021). In *Arabidopsis*, AtbZIP60 responds to endoplasmic reticulum stress through the IRE1-bZIP60 mRNA splicing pathway (Deng et al., 2011). Briefly, AtIRE1 selectively recognizes and cleaves the unspliced *bZIP60* mRNA that normally exists in the ER membrane, and the resulting spliced *bZIP60* mRNA can be translated into an active bZIP transcription factor (Howell, 2013). The subcellular localization experiment conducted in the current study demonstrated that SpbZIP60 is a nuclear protein, but whether this means SpbZIP60 contributes to the ER stress response remains to be determined. At present, there are relatively few studies on Cd-mediated ER stress in plants.

## Conclusion

In this study, we identified 92 bZIP genes in *S. plumbizincicola* and analyzed their evolutionary relationships. These genes were divided into 12 subgroups, and the members of each subgroup had common gene structures and motif compositions. An analysis of the *S. plumbizincicola* bZIP genes revealed eight segmental duplication events, but no tandem duplication events, suggesting that segmental duplication events were the main force driving the evolution of the bZIP gene family in *S. plumbizincicola.* A collinearity analysis involving *S. plumbizincicola* and other species and a comparison between the *S. plumbizincicola* genes and the genes encoding bZIP transcription factors with known functions in model plants will provide new clues regarding SpbZIP functions. We also characterized the *SpbZIP* expression profiles under Cd stress conditions and constructed a co-expression network comprising 11 *SpbZIP* hub genes. The results of this study reflect the importance of SpbZIP transcription factors for regulating plant responses to Cd stress. The expression of the hub gene *SpbZIP60* was induced by Cd stress and enhanced the Cd tolerance of transgenic *Arabidopsis*. Overall, these findings may provide new insights into the stress response-related functions of SpbZIP transcription factors in *S. plumbizincicola*.

## Data Availability Statement

The original contributions presented in the study are included in the article/Supplementary Material, further inquiries can be directed to the corresponding author/s.

## Author Contributions

ZL and RZ designed the experiments. ZL performed the experiments, analyzed the data, and wrote the manuscript. KJ and MY analyzed the data and prepared the display items. ZL, WQ, XJH, and RZ helped revise the manuscript. LW and CW designed the work and provided materials. XYH provided the culture room. All authors read and approved the final manuscript.

## Conflict of Interest

The authors declare that the research was conducted in the absence of any commercial or financial relationships that could be construed as a potential conflict of interest.

## Publisher’s Note

All claims expressed in this article are solely those of the authors and do not necessarily represent those of their affiliated organizations, or those of the publisher, the editors and the reviewers. Any product that may be evaluated in this article, or claim that may be made by its manufacturer, is not guaranteed or endorsed by the publisher.

## Footnotes

1
http://pfam.xfam.org/

2
http://www.arabidopsis.org/

3
http://smart.embl-heidelberg.de/

4
https://www.ncbi.nlm.nih.gov/Structure/cdd/wrpsb.cgi

5
https://web.expasy.org/protparam/

6
http://psort1.hgc.jp/form.html

7
https://itol.embl.de/

8
http://bioinformatics.psb.ugent.be/webtools/plantcare/html/

9
https://github.com/CJ-Chen/TBtools

10
http://gsds.cbi.pku.edu.cn/

11
http://meme-suite.org/tools/meme

12
http://circos.ca/
