# Supplementary material for: Identification and Analysis of bZIP Family Genes in Sedum plumbizincicola and Their Potential Roles in Response to Cadmium Stress
Source: Front Plant Sci. 2022 Apr 27;13:859386. doi: 10.3389/fpls.2022.859386 (PMC9094143; doi:10.3389/fpls.2022.859386)
Supplement: Supplementary file 1 [file Data_Sheet_1.zip › Figure S1.DOCX]

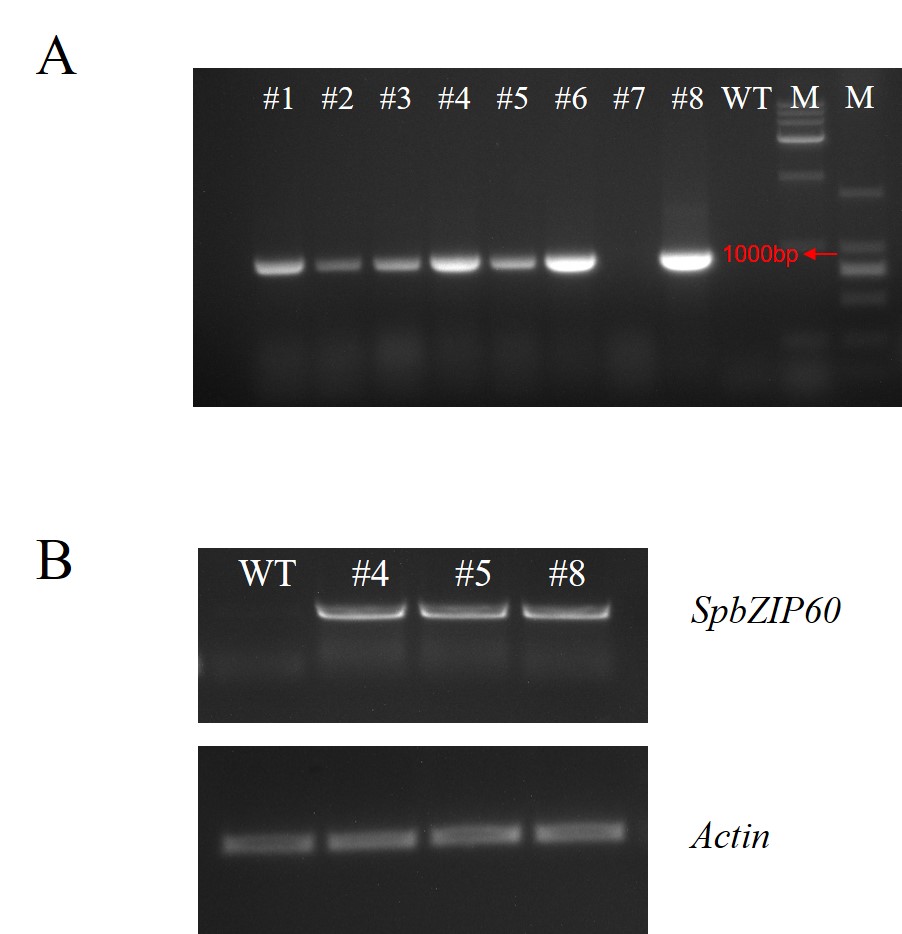


**Supplementary Figure 1.** (A) Eight T_1_ transgenic lines were verified by PCR using genomic DNA templates. (B) Semi-RT-PCR analysis of *SpbZIP60* expression levels in wild-type and three overexpression lines. *AtActin* was used as the internal control.
